# Rekombinante Allergene, Peptide und *Virus-like Particles* in der Immuntherapie von Allergien

**DOI:** 10.1007/s00103-020-03231-7

**Published:** 2020-10-23

**Authors:** Thomas Holzhauser, Frank Schuler, Simone Dudek, Susanne Kaul, Stefan Vieths, Vera Mahler

**Affiliations:** Paul-Ehrlich-Institut, Bundesinstitut für Impfstoffe und Biomedizinische Arzneimittel (PEI), Paul-Ehrlich-Straße 51–59, 63225 Langen, Deutschland

**Keywords:** Rekombinante Allergene, Peptide, VLP, AIT, Regulation, Recombinant allergens, Peptides, VLP, AIT, Regulation

## Abstract

Aktuell werden extraktbasierte Therapieallergene aus natürlichen Allergenquellen (z. B. Hausstaubmilben, Baum- und Gräserpollen) zur allergenspezifischen Immuntherapie (AIT) eingesetzt, dem einzigen kausalen Therapieansatz, der durch Toleranzentwicklung allergische Erkrankungen positiv verändern und langfristig eine Krankheitsprogression verhindern kann. Aufgrund von Schwankungen in der natürlichen Zusammensetzung der Ausgangsmaterialien und unterschiedlichen Herstellungsprozessen ergeben sich Varianzen in Proteingehalt, Allergenkomposition und allergener Aktivität gleichlautender Produkte, was spezifische Herausforderungen an ihre Standardisierung stellt. Die Aufklärung der Nukleotidsequenzen allergieauslösender Proteine führte zur Entwicklung molekularer AIT-Ansätze. Dies ermöglicht die Applikation von ausschließlich allergologisch relevanten Strukturen und schließt chemisch synthetisierte Peptide, rekombinante Einzelallergene und Therapiemoleküle mit hypoallergenen Eigenschaften ein, die potenziell eine Auftitration mit höheren Allergendosen ohne allergische Nebenwirkungen und schnelleres Erreichen der wirksamen kumulativen Dosis ermöglichen. Zudem lassen sich weitere Modifikationen der AIT-Ansätze zur Verbesserung der allergenen und immunogenen Eigenschaften, wie zum Beispiel durch Verwendung von *Virus-like Particles* (VLP), erreichen. Bis dato sind die hier beschriebenen AIT-Ansätze ausschließlich in der klinischen Erprobung. Dieser Artikel gibt eine Übersicht über die publizierten, in klinischen AIT-Studien eingesetzten molekularen Allergietherapeutika. Ihr Mehrwert sowie Herausforderungen gegenüber etablierten Therapieallergenen werden diskutiert. Ziel ist die Entwicklung hochwirksamer und verträglicher AIT-Präparate mit einer verbesserten Patientenakzeptanz und -adhärenz.

## Abstract (en)

Currently, extract-based therapeutic allergens from natural allergen sources (e.g., house dust mites, tree and grass pollen) are used for allergen-specific immunotherapy (AIT), the only causative therapy that can exhibit positive disease-modifying effects by tolerance induction and prevention of disease progression. Due to variations in the natural composition of the starting materials and different manufacturing processes, there are variations in protein content, allergen composition, and allergenic activity of similar products, which poses specific challenges for their standardization. The identification of the nucleotide sequences of allergenic proteins led to the development of molecular AIT approaches. This allows for the application of exclusively relevant structures as chemically synthesized peptides, recombinant single allergens, or molecules with hypoallergenic properties that potentially allow for an up-dosing with higher allergen-doses without allergic side effects leading more quickly to effective cumulative doses. Further modifications of AIT preparations to improve allergenic and immunogenic properties may be achieved, e.g., by including the use of virus-like particles (VLPs). To date, the herein described therapeutic approaches have been tested in clinical trials only. This article provides an overview of published molecular approaches for allergy treatment used in clinical AIT studies. Their added value and challenges compared to established therapeutic allergens are discussed. The aim of these approaches is to develop highly effective and well-tolerated AIT preparations with improved patient acceptance and adherence.

## Einleitung

Allergische Erkrankungen sind Überempfindlichkeitsreaktionen des Immunsystems und betreffen bis zu 30 % der Bevölkerung industrialisierter Länder. Sie stellen neben dem individuellen Leiden und Verlust an Lebensqualität eine hohe sozioökonomische Belastung dar [1]. Häufig werden Allergien durch Immunglobulin E (IgE) vermittelt [2]. Sie weisen unterschiedliche klinische Manifestationen auf, wie beispielsweise allergische Rhinokonjunktivitis, atopische Dermatitis, Urtikaria, Angioödem, allergisches Asthma und systemische allergische Reaktionen bis hin zum anaphylaktischen Schock [1]. Weitverbreitete Auslöser dieser Allergien vom IgE-vermittelten Soforttyp („Typ I“) sind Baum‑, Gräser- und Kräuterpollen, Tierepithelien, Insektengifte, Hausstaub- und Vorratsmilben, Schimmelpilze, Nahrungsmittel und Arzneimittel. Darüber hinaus sind zahlreiche Typ-I-Allergene von beruflicher Bedeutung (z. B. Mehlstäube, Holzstäube, Enzyme, Naturgummilatex; [3]). Die molekularen Auslöser sind überwiegend Proteine (Allergene), die nur bei bereits sensibilisierten Personen nach Haut- und Schleimhautkontakt, Inhalation, Injektion oder Ingestion allergische Reaktionen auslösen können. Auftretende Symptome können medikamentös supprimiert werden; diese symptomatischen Therapieansätze modulieren die Grundkrankheit nicht. Dagegen bewirkt die allergenspezifische Immuntherapie (AIT), als einzige kausale Therapieform, durch Modulation des Immunsystems eine allergenspezifische Toleranzentwicklung und infolgedessen Linderung der Symptome und damit einhergehend Reduktion der Medikation. Langfristig kann auch eine Krankheitsprogression, z. B. der sogenannte Etagenwechsel von der allergischen Rhinokonjunktivitis hin zum allergischen Asthma, verhindert werden [4]. Verschiedene komplexe Mechanismen der AIT werden je nach Therapieansatz beschrieben (s. unten; [5]).

Die derzeit marktfähigen, in der klinischen Versorgung routinemäßig eingesetzten AIT-Präparate werden weltweit ausschließlich aus natürlichen Allergenquellen, im Wesentlichen durch Extraktion des Gesamtproteinanteils einschließlich der therapiewirksamen allergenen Proteine gewonnen. Aufgrund des natürlichen Ausgangsmaterials unterliegen diese AIT-Präparationen Schwankungen in ihrer Komposition (s. unten). Hohe Ansprüche an die Standardisierung sind daher notwendig, um eine angemessene und konsistente Chargenqualität der Allergenprodukte zu gewährleisten [6, 7].

Therapierelevante Allergene können auch biotechnologisch oder chemisch-synthetisch in hoher definierter Qualität hergestellt werden. Dies wurde durch die Identifizierung der einzelnen Allergenkomponenten und die Aufklärung ihrer codierenden Desoxyribonukleinsäuresequenzen (komplementäre DNA, cDNA) möglich. Seit der biotechnologischen Herstellung der ersten rekombinanten Allergene Ende der 1980er-Jahre wurden bislang etwa 1000 Allergenkomponenten entschlüsselt und mit einheitlicher Nomenklatur registriert [8]. Die grundlegende Kenntnis dieser allergenen Strukturen und die Verfügbarkeit von rekombinanten Allergenen mittels Gentechnologie ermöglichte

- Verbesserungen der Standardisierung von traditionellen Allergenextrakten [9],
- Verbesserungen der In-vitro-Diagnostik im Sinne einer *Component Resolved Diagnosis* (bezogen auf klinische Situation und Risiko schwerer Reaktionen), welche die extraktbasierte Diagnostik ergänzt [10],
- die Identifizierung therapierelevanter Allergene (sowohl für molekulare Therapieansätze als auch für extraktbasierte Produkte), auch im Sinne einer personalisierten Medizin, sowie
- die gentechnische Modifikation der Allergene (hypoallergene Strukturen, Hybridstrukturen, Design von Carrier-Systemen) mit optimierten allergenen und immunogenen Eigenschaften.

Trotz einer Vielzahl klinischer Studien zum Konzeptnachweis stehen rekombinante oder chemisch-synthetische Allergenpräparationen bisher nicht als zugelassene Allergentherapeutika zur Verfügung (s. Beitrag von Mahler et al. in diesem Themenheft). Im Folgenden werden diese Ansätze näher erläutert und der potenzielle Mehrwert sowie die Herausforderungen bei diesen innovativen Allergietherapeutika im Vergleich zu den marktüblichen extraktbasierten AIT-Präparationen diskutiert.

## Von Allergenextrakten zu definierten Therapiemolekülen

Herausforderungen bei traditionellen extraktbasierten Allergenprodukten sind Varianzen in der Protein- und Allergenzusammensetzung aufgrund von natürlichen Schwankungen der Ausgangsmaterialien oder Unterschieden in den Produktionsschritten verschiedener Hersteller [6, 7]. Dies kann zu Schwankungen innerhalb unterschiedlicher Chargen desselben Produktes eines Herstellers führen oder zu genereller Nichtvergleichbarkeit der Produkte unterschiedlicher Hersteller zur Behandlung derselben Allergie, z. B. durch Unterschiede im Gehalt der relevanten Allergene oder der allergenen Gesamtaktivität (Potenz). Zudem weisen manche Extrakte (z. B. Hausstaubmilben oder Schimmelpilze) eine endogene Proteaseaktivität auf, sodass es zum Abbau von Allergenkomponenten während der Herstellung und Lagerung kommen kann. Diese Parameter bedürfen deshalb einer besonderen Beachtung, um eine angemessene und konsistente Chargenqualität zu gewährleisten. Die staatliche Chargenprüfung dient dazu, derartige Schwankungen innerhalb der Chargen eines Herstellers in engen Grenzen zu halten.

**Figure 1:**
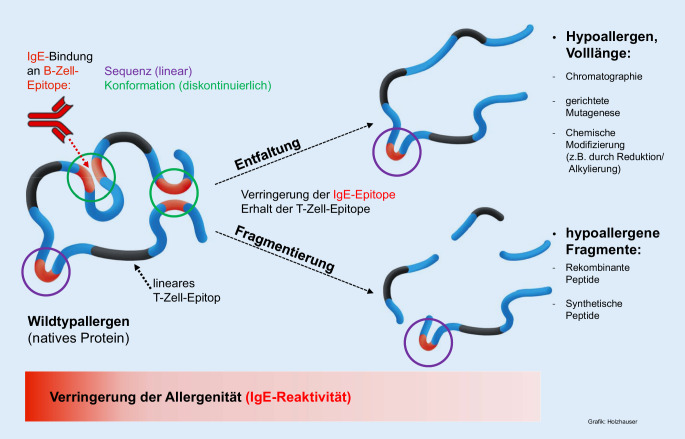

Auch sind Neusensibilisierungen gegen bislang allergologisch nicht relevante Strukturen in extraktbasierten Gemischen prinzipiell möglich [11]. Viele dieser Herausforderungen können mit hochreinen, biotechnologisch oder synthetisch hergestellten Therapieallergenen vermieden werden, die bei geeigneter Auswahl ausschließlich therapeutisch relevante Strukturen enthalten und mit hoher Reinheit, definierter Aminosäurezusammensetzung, akkurater Menge und exakter physikalisch-chemischer Charakterisierung sowie eng gesetzten Spezifikationen hergestellt werden können (Tab. 1). Doch auch zu solchen Produkten gibt es offene Fragen: So ist zum Beispiel die Bedeutung von natürlich vorkommenden Isoformen und Sequenzvarianten der Allergene für die Wirksamkeit und Sicherheit der AIT ungeklärt [12]. Ein Nachteil von nichtmodifizierten Allergenprodukten sind potenziell auftretende unerwünschte IgE-vermittelte allergische Reaktionen, welche mittels protrahierter Aufdosierung beginnend mit sehr geringen Allergendosen minimiert werden [4, 13]. Während Extrakte durch chemische Quervernetzung oder Reduktion und Alkylierung relativ ungerichtet modifiziert werden können, um die Häufigkeit allergischer Reaktionen bei der Applikation zu vermindern, können auf molekularer Ebene gezielte Änderungen der immunologischen und allergenen Eigenschaften rekombinanter bzw. chemisch-synthetischer Allergenpräparationen herbeigeführt werden (Abb. 1). Durch erzwungene Entfaltung, beispielsweise durch gezielte Aminosäuresubstitution, chemische Modifizierung (z. B. durch Reduktion/Alkylierung) oder chromatographische Umfaltung sowie durch eine definierte Fragmentierung entstehen sogenannte hypoallergene Strukturen mit verringerter IgE-Reaktivität aufgrund des Verlustes von faltungsabhängigen Epitopen (Konformationsepitopen) und folglich erhöhter Tolerabilität. Lediglich konformationsunabhängige, lineare Epitope bleiben zur IgE-Bindung erhalten. Es wird diskutiert, dass dadurch höhere Dosen appliziert werden können und eine wirksame kumulative Dosis schneller erreicht werden kann [14]. Zudem können Hybridmoleküle aus allergenen Strukturen und molekularen Adjuvanzien sowie *Virus-like Particles* (VLP) oder anderen funktionalen Elementen, wie beispielsweise Translokationsmotiven (z. B. zur zytoplasmatischen Aufnahme extrazellulärer Proteine), mit optimierten immunologischen und damit therapeutischen Eigenschaften generiert werden [15, 16]. Die meisten der letztgenannten Ansätze befinden sich jedoch bisher auf einem experimentellen präklinischen Entwicklungsstand.

**Table 1:** 

rAllergene, rVLP, synthetische Peptide		Traditionelle extraktbasierte Allergenprodukte	
Mehrwert	Herausforderungen	Mehrwert	Herausforderungen
AIT ausschließlich mit allergologisch relevanten Strukturen	Selektion der allergologisch relevanten Struktur(en); mögliches Fehlen therapierelevanter Allergene (oder deren Isoformen)	AIT mit einer natürlichen Mischung aus Allergenen und deren Isoformen	Mögliches Fehlen therapierelevanter Allergene (oder Isoformen) oder proteolytische Allergendegradation; mögliche Kreuzkontamination mit anderen natürlichen Allergenquellen
Definierte Moleküle von definierter Qualität (GMP): hohe Reinheit, definierte Menge, reproduzierbare Qualität	Rekombinante Allergentherapeutika: Etablierung einer GMP-Produktion in gentechnischen Anlagen	Einfache und etablierte Herstellung der Allergenextrakte	Standardisierungsbedarf: natürliche Schwankungen in Allergenzusammensetzung sowie Protein- und Allergengehalt; genaue Protein- bzw. Allergenzusammensetzung meist unbekannt
Definierte Modifikation des immunologischen und allergenen Potenzials vielfältig möglich	Rekombinante Proteine: Ausschließlich zentralisiertes Zulassungsverfahren	Etablierte Marktautorisierung aufgrund jahrzehntelanger Erfahrung mit Allergenextrakten; Marktautorisierung über nationale, zentrale, dezentrale oder MR-Zulassungsverfahren möglich	Modulation des immunologischen und allergenen Potenzials nur eingeschränkt möglich
Vermeidung von möglichen Neosensibilisierungen	Bezogen auf Marktetablierung (nicht bezüglich Zulassung): Demonstration eines Mehrwerts im Vergleich zu etablierten und in der klinischen Praxis vorhandenen Allergenextrakten		Mögliche Neosensibilisierungen gegen bislang nicht relevante allergene Strukturen oder unerwünschte allergene Kreuzkontaminationen aus natürlichen Quellen

*AIT* allergenspezifische Immuntherapie, *GMP* gute Herstellungspraxis (engl. „good manufacturing practice“), *MR* gegenseitige Anerkennung (engl. „mutual recognition“)

Je nach Therapieansatz kommen unterschiedliche Mechanismen der insgesamt sehr komplexen Wirkungsweise der AIT zum Tragen [5]. Diese schließen die Generierung allergenspezifischer regulatorischer T‑ und B‑Zellen sowie die Regulation von spezifischem allergievermittelnden IgE und allergenblockierendem IgG4 ein. Hierbei sind allergenspezifische Th2- und Th1-Zellen beteiligt, die herab- bzw. aufreguliert werden. Die Aktivierung inhibitorischer Fcγ-Rezeptoren als möglicher Mechanismus wird diskutiert. Des Weiteren sind angeborene lymphoide Zellen und zytotoxische T‑Zellen involviert. Aus diesem Zusammenspiel ergibt sich eine Veränderung hin zu einer T‑Zelltoleranz und Reduzierung der Reaktivität allergischer Effektorzellen wie Basophilen und Mastzellen [5, 17]. Unter anderem wird die Generierung von allergenblockierenden IgG-Antikörpern bei (fast) allen Ansätzen als ein möglicher Wirkmechanismus erwartet, wobei ihre variable Induktion – möglicherweise in Abhängigkeit von der Peptidlänge – und ihre Bedeutung für die Wirkmechanismen von T‑zellpeptidbasierten AIT-Ansätzen noch nicht abschließend geklärt ist [18].

**Figure 2:**
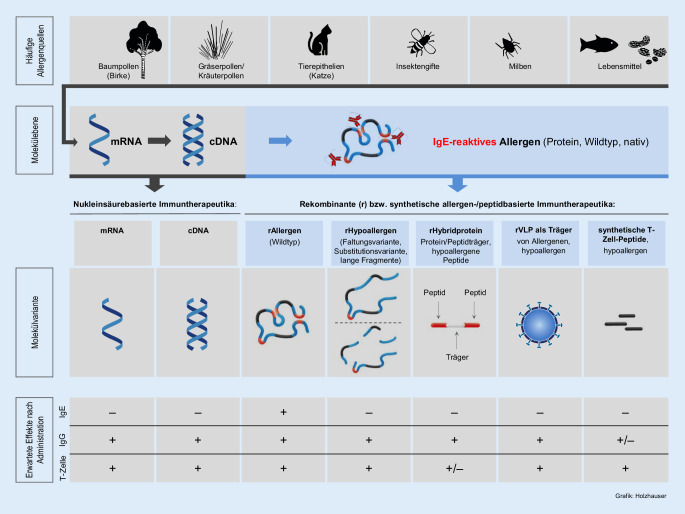

Abb. 2 verdeutlicht schematisch die Vielfalt an möglichen innovativen Allergietherapeutika ausgehend von der allergencodierenden *Messenger-*RNA (mRNA) bzw. cDNA, die prinzipiell direkt für einen immuntherapeutischen Ansatz genutzt werden können. In Abhängigkeit von den molekularen Eigenschaften der jeweiligen Ansätze werden die erwarteten immunologischen Effekte schematisch dargestellt bezüglich Allergenität (Bindung und Kreuzvernetzung von allergenspezifischem IgE), Antigenität (Bildung von blockierenden allergenspezifischen IgG) und Beteiligung von allergenspezifischen T‑Zellen. Das Prinzip der nukleinsäurebasierten AIT wurde bisher nur in einzelnen klinischen Studien eingesetzt und wird deshalb hier nicht weiter adressiert [19]. Neben den rekombinanten nativ gefalteten („Wildtyp“-)Allergenen, die in der Allergenität (insbesondere IgE-Reaktivität) ihren natürlichen Pendants entsprechen, wurden rekombinante Hypoallergene, Hybridproteine und VLP sowie synthetische T‑Zellpeptide in klinischen AIT-Studien untersucht. Bis auf die rekombinanten Wildtypallergene zielen alle anderen Ansätze auf eine reduzierte IgE-Reaktivität und damit ein erwartetes geringeres Auftreten von allergischen Reaktionen nach Applikation ab, was aber nicht immer den Ergebnissen klinischer Untersuchungen entspricht [20].

Tab. 2 gibt eine summarische Übersicht über publizierte klinische Studien (ohne Anspruch auf Vollständigkeit) mit rekombinanten oder chemisch-synthetischen Molekülen und modifizierten Strukturen in der AIT von inhalativen saisonalen (Birkenpollen, Gräserpollen), inhalativen perennialen (Katzenepithelien, Hausstaubmilben) sowie ingestiven perennialen (Apfel, Erdnuss, Fisch) Allergien. Die folgenden Abschnitte erläutern schwerpunktmäßig die Erkenntnisse aus den Publikationen der in Tab. 2 genannten durchgeführten klinischen Studien. Darüber hinaus gibt es eine Vielzahl von publizierten präklinischen Arbeiten, oftmals im murinen Allergiemodell, die aufgrund der Fokussierung dieses Artikels auf klinische Aspekte nicht adressiert werden.

**Table 2:** 

Allergie	Therapiemoleküle (IMP) (Molekülvarianten, Identität, Formulierung)	Studien (Applikation, Design, Identifikationsnummer *NCT* Number) in Datenbank (*ClinicalTrials.gov, Sponsor*)	Referenzen
*Rekombinante Allergene, Hypoallergene und Hybridproteine*			
Birkenpollen	rBet v 1 Hauptallergen (Volllänge, Wildtyp, in Lösung); direkter Vergleich mit Birkenpollenextrakt	SCIT, bis Phase II, DBPC, NCT00410930,* Stallergènes, FRA*	[11]
Birkenpollen	rBet v 1 Hauptallergen (Volllänge, Wildtyp, Tablette)	SLIT, bis Phase II, DBPC, NCT00901914, NCT00396149, NCT00889460, *Stallergènes, FRA*	[22]
Apfel	rMal d 1 Hauptallergen (Volllänge, Wildtyp, in Lösung); direkter Vergleich mit rBet v 1 (Volllänge, Wildtyp, in Lösung)	SLIT, explorativ, DBPC, NCT01449786, *Medizinische Universität Wien, AUT*	[23, 24]
Wiesenlieschgras	rPhl p 1, r Phl p 2, rPhl p 5a + b, rPhl p 6 Allergenmischung (Volllänge, Wildtyp, adsorbiert an Alum.)	SCIT, bis Phase III, DBPC, NCT00309036, NCT00671268, NCT01353755, NCT00666341, EudraCT2007-02808-18, *Allergopharma, DE*	[25, 26]
Birkenpollen	rBet v 1 Hauptallergen (Faltungsvariante, hypoallergen, adsorbiert an Alum.); direkter Vergleich mit Birkenpollendepotextrakt	SCIT, bis Phase III, DB und DBPC, NCT00266526, NCT00309062, NCT00554983, NCT00841516, NCT01490411, *Allergopharma, DE*	[27, 28]
Birkenpollen	rBet v 1 Hauptallergen (Volllängen-Trimer und Fragmente, hypoallergen, adsorbiert an Alum.)	SCIT, bis Phase II, DBPC, kein NCT-Eintrag	[29, 30]
Katzenepithelien	Hybridprotein aus rFel d 1 Hauptallergen und Translokationssequenz (TAT) als modularer Antigentransporter (MAT-rFel d 1: Volllänge, hypoallergen, adsorbiert an Alum.)	ILIT, bis Phase I/IIa, DBPC, NCT00718679, *Universität Zürich, CH*	[31]
Wiesenlieschgras	Hybridprotein aus Peptiden/Epitopen der Graspollenallergene Phl p 1, Phl p 2, Phl p 5 und Phl p 6, und HBV PreS als Träger (BM32: synthetische B‑zellreaktive Peptide, carrier-gebunden, hypoallergen, adsorbiert an Alum.)	SCIT, bis Phase IIb, DBPC, NCT01350635, NCT01445002, NCT01538979, NCT02643641 (Hepatitis B: NCT03625934), *Biomay, AUT (Viravaxx, AUT)*	[32, 33]
Fisch	rCyp c 1 Hauptallergen (mCyp c 1: Volllänge, Substitutionsvariante, hypoallergen)	SCIT, bis Phase IIa/b, DBPC, NCT02017626, NCT02382718, *Rigshospitalet, DK*	[34–37]
Erdnuss	rAra h 1, rAra h 2, rAra h 3 Allergenmischung (EMP1,2,3: Volllänge, Substitutionsvarianten, hypoallergen, gekapselt mit inaktivierten *E. coli*)	Rektal, Phase I, non-DBPC, NCT00850668, *Allertein Therapeutics, USA*	[38]
*Rekombinante Virus-like Particles*			
Hausstaubmilbe	QbG10 VLPs bepackt mit Typ A CpG (CYT003-QbG10), **ohne** Allergen	SCIT, bis Phase IIb, DBPC, NCT00800332, *Cytos Biotechnology, CH*	[40]
Allergisches Asthma	QbG10 VLPs bepackt mit Typ A CpG (CYT003-QbG10), **ohne** Allergen	SCIT, bis Phase II, DBPC, NCT00890734, *Cytos Biotechnology, CH*	[41]
Hausstaubmilbe	Bakteriophagen Qβ-VLP bepackt mit G10 CpG als Adjuvans (QbG10), gemischt mit Hausstaubmilbenextrakt (Volllänge, Wildtyp, adsorbiert an Alum.)	SCIT, bis Phase I/IIa, NCT00652223, *Cytos Biotechnology, CH*	[42]
Hausstaubmilbe	Bakteriophagen Qβ-VLP chemisch gekoppelt mit B‑Zellepitop (21 Aminosäuren) des Hauptallergens Der p 1 (Qβ-Der p 1: mit prokaryotischer RNA beladen, hypoallergen, in Lösung)	SCIT, IMIT, bis Phase I, kein NCT-Eintrag	[43]
*Synthetische Peptide*			
Birkenpollen	3 überlappende Peptide des Hauptallergens Bet v 1 (AllerT: Fragmentlänge 49–71 Aminosäuren, hypoallergen, adsorbiert an Alum.)	SCIT, bis Phase IIb, DBPC, NCT01719133, NCT01720251, NCT02143583, NCT02271009, NCT02943720, *Anergis, CH*	[45–48]
Katzenepithelien	2 immunodominante T‑Zellepitoppeptide des Hauptallergens Fel d 1 (AllervaxCAT: je 27 Aminosäuren, hypoallergen, in Lösung)	SCIT, bis Phase III, DBPC, kein NCT-Eintrag, *ImmuLogic Pharmaceutical, USA*	[49, 50]
Katzenepithelien	7 kurze Peptide, immunregulatorische T‑Zellepitope (SPIRE) des Hauptallergens Fel d 1 (ToleroMuneCat, CatPAD: 13-17 Aminosäuren, hypoallergen, in Lösung)	IDIT, bis Phase III, DBPCFC, NCT01620762, NCT01604018, NCT02040844, NCT02311413, *Circassia, UK*	[20, 51–53]
Gräserpollen	7 kurze Peptide, immunregulatorische T‑Zellepitope (SPIRE) der konservierten Gruppe 1/4/5 Grasallergene (ToleroMune Grass; 10–18 Aminosäuren, hypoallergen, in Lösung)	IDIT, bis Phase II, DBPC, NCT01385800, *Circassia, UK*	[54]

*AIT* Allergenspezifische Immuntherapie, *SCIT* subkutane Immuntherapie, *SLIT* sublinguale Immuntherapie, *ILIT* intralymphatische Immuntherapie, *IDIT* intradermale Immuntherapie, *IMIT* intramuskuläre Immuntherapie, *DB* doppelblind, *PC* placebokontrolliert, *VLP* Virus-like Particles, *IMP* Prüfpräparat („investigational medicinal product“), *Alum.* Aluminiumhydroxiddepot

## Rekombinante Allergene und Derivate

Rekombinante Proteine werden auf Basis ihrer cDNA mittels Genvektoren wie Plasmide in pro- oder eukaryotische Wirtszellen (z. B. *Escherichia coli* oder Hefen) eingebracht, wo sie durch den Biosyntheseapparat der Wirtszelle exprimiert werden. Im Anschluss an den Fermentations- und Expressionsprozess erfolgt eine – zumeist – chromatographische Reinigung und umfangreiche physikochemische und immunologische Charakterisierung. Einen vereinfachten Überblick über den Produktionsprozess rekombinanter Allergene als Therapiepräparate geben Nandy und Kollegen [21]. Die in Tab. 2 zusammengefassten Studien wurden ausschließlich mit in *E. coli* heterolog exprimierten Proteinen durchgeführt. Die nachfolgende Darstellung der AIT-Präparate entspricht der Reihenfolge von Tab. 2 und wird weiter durch die Schematisierung in Abb. 2 verdeutlicht.

Rekombinante Volllängenallergene mit einer dem Wildtypallergen vergleichbaren nativen Faltung wurden zur AIT der Birkenpollenallergie, der Wiesenlieschgrasallergie und der birkenpollenassoziierten Apfelallergie in klinischen Studien untersucht (Tab. 2). In einer placebokontrollierten subkutanen Phase-II-Studie (SCIT) mit Birkenpollenallergikern wurde die Sicherheit und Wirksamkeit von rekombinantem Hauptallergen Bet v 1 der Birkenpollen (rBet v 1), mit gereinigtem natürlichen Bet v 1 (nBet v 1) und mit Birkenpollenextrakt mit gleichem Schema der Aufdosierung und Dosierhaltung über einen Verlauf von 2 Jahren untersucht [11]. Als primärer Endpunkt wurden jeweils der tägliche durchschnittliche Symptom-Score und der Medikations-Score während der Pollensaison ausgewertet. In allen aktiven Gruppen (rBet v 1, nBet v 1, Extrakt) zeigte sich eine vergleichbare signifikante Verbesserung gegenüber Placebo, bezogen auf die Rhinokonjunktivitissymptome (ca. 50 %), eine Reduzierung der Medikation (ca. 60–70 %) und der Hautreaktivität. Alle aktiven Gruppen wiesen einen deutlichen Anstieg von allergenspezifischem IgG auf, welcher mit rBet v 1 tendenziell am höchsten ausfiel. Auftretende unerwünschte Reaktionen während der AIT waren in den aktiven Gruppen vergleichbar. Es gab keine schweren oder systemischen Reaktionen mit rBet v 1. In 10 % (3/29 Patienten) der mit Birkenpollenextrakt behandelten Gruppe, nicht aber in den r/nBet v 1- behandelten Gruppen (32 bzw. 29 Patienten) traten neue Sensibilisierungen (IgE-Spezifitäten) gegen Bet v 2, einem allergenen Profilin, auf. Birkenpollenextrakt, rBet v 1 und nBet v 1 unterschieden sich in dieser Studie nur hinsichtlich hervorgerufener Neusensibilisierungen.

In einer anderen placebokontrollierten Phase-II-Studie mit Birkenpollenallergikern wurde Wildtyp rBet v 1 sublingual (SLIT) als Tablette in 3 verschiedenen Dosen über 5 Monate täglich verabreicht [22]. Als primärer Endpunkt wurde der durchschnittliche bezüglich der eingenommenen Medikation adjustierte Symptom-Score während der Birkenpollensaison gewählt. Dieser Score war gegenüber Placebo signifikant reduziert. Die Autoren beschrieben die rBet v 1- Tablette als gut toleriert, in Übereinstimmung mit dem bekannten Sicherheitsprofil der SLIT, wobei lokale Symptome in Mund und Rachen während der Behandlung auftraten. Eine wesentliche Schlussfolgerung aus dieser Studie war, dass rekombinante Allergene *per se *in dem gewählten Studiendesign unter Berücksichtigung der Power der Studie keine ausgeprägteren Effekte erzielten als Allergenextrakte.

Eine explorative placebokontrollierte SLIT-Studie mit birkenpollenassoziierten Apfelallergikern zeigte, dass das rekombinante Hauptallergen von Apfel, rMal d 1, nicht aber das primär sensibilisierende Birkenpollenhauptallergen Bet v 1, eingesetzt in Form von rBet v 1, die klinische Symptomatik der Mal d 1- vermittelten Apfelallergie signifikant reduzieren konnte [23, 24]. Die Autoren beschrieben eine gute Verträglichkeit ohne systemische oder schwere Reaktionen bei 4‑monatiger sublingualer Gabe von rMal d 1.

Während bei der Birkenpollenallergie und assoziierten Nahrungsmittelallergien das Hauptallergen Bet v 1 bzw. die homologen Bet v 1- assoziierten Allergene, wie Mal d 1 beim Apfel, den überwiegenden Anteil an der allergenen Potenz haben, sind bei Gräserpollenallergien verschiedene Allergene relevant. Um dem Rechnung zu tragen, wurden in einer placebokontrollierten AIT-Studie mit Gräserpollenallergikern 5 rekombinante Allergene aus Wiesenlieschgraspollen (rPhl p 1, 2, 5a, 5b, 6) an Aluminiumhydroxid adsorbiert und über einen Zeitraum von etwa 1 ½ Jahren subkutan injiziert [25, 26]. Die rekombinanten Allergene wurden allgemein gut toleriert mit milden lokalen Reaktionen an den Injektionsstellen, aber auch gelegentlichen systemischen Reaktionen, vergleichbar denen bei extraktbasierten Studien. Primärer Endpunkt war ein kombinierter Symptom-Medikations-Score während der Graspollensaison. Insgesamt wurde von einer signifikanten Verbesserung der Rhinokonjunktivitissymptome gegenüber Placebo berichtet. Die Verbesserung der klinischen Symptomatik war begleitet durch einen Anstieg von allergenspezifischem IgG.

Zur Verbesserung der Tolerabilität und Verkürzung der Therapiedauer wurden rekombinante Hypoallergene, hypoallergene Fragmente und Hybridmoleküle zur Immuntherapie von Allergien gegen Birkenpollen, Gräserpollen, Katzenepithelien sowie Nahrungsmittelallergien (Erdnuss, Fisch) entwickelt (Tab. 2).

Eine hypoallergene rekombinante Faltungsvariante von Bet v 1, rBet v 1-FV, adsorbiert an Aluminiumhydroxid, wurde Birkenpollenallergikern über 10 Wochen wöchentlich subkutan injiziert und die Wirkung (Symptom-Score) durch Provokation mit Birkenpollen in einer Expositionskammer vor/nach der Behandlung ermittelt. In dieser Dosisfindungsstudie zeigte sich gegenüber Placebo eine signifikante Verbesserung der Rhinokonjunktivitissymptomatik bei gleichzeitig guter Tolerabilität [27]. In einer weiteren komparativen SCIT-Studie erhielten Birkenpollenallergiker präsaisonal entweder 8 wöchentliche Injektionen mit rBet v 1-FV oder 14-wöchentliche Injektionen mit einem etablierten Depotbirkenpollenextrakt. Nach der präsaisonalen Behandlung lag der kombinierte Symptom-Medikations-Score während der 3‑wöchigen Birkenpollensaison für rBet v 1-FV-Behandelte etwa bei der Hälfte des Wertes im Vergleich zu dem der mit Extrakt behandelten Allergiker. Nach 2 Jahren ergaben sich für rBet v 1-FV-Behandelte keine signifikanten Unterschiede in der Verbesserung der Symptomatik im direkten Vergleich zum Birkenpollenextraktpräparat. rBet v 1-FV konnte dabei in höheren Dosen als das native Protein ohne Anstieg von unerwünschten Reaktionen verabreicht werden [28]. Mit diesem Produkt wurde nachfolgend eine Phase-III-Studie durchgeführt (NCT00554983), deren Ergebnisse nicht publiziert wurden. Das Entwicklungsprogramm wurde nicht weitergeführt.

Ein anderer hypoallergener Ansatz wurde durch Trimerisierung bzw. Fragmentierung von rBet v 1 untersucht [29, 30]. Gegenüber Placebo zeigten in einer Phase-II-Studie die aktiv behandelten Birkenpollenallergiker im kombinierten Symptom-Medikations-Score während der Birkenpollensaison einen Trend zur Verbesserung, allerdings ohne statistische Signifikanz.

Zur AIT der Katzenhaarallergie wurde Patienten ein rekombinantes an Aluminiumhydroxid adsorbiertes Hybridprotein (MAT-rFel d 1) in einer Phase-II-Studie intralymphatisch appliziert. Dieses wurde aus dem Hauptallergen der Katze (Fel d 1) und einer Translokationssequenz, fungierend als modularer Antigentransporter (MAT), hergestellt [31]. In der placebokontrollierten AIT-Studie konnte mit nur dreimaliger intralymphatischer Injektion eine bis zu 74-fach größere nasale Toleranz im Vergleich zu einer etwa dreifachen Verbesserung durch Placebo erreicht werden. Eine Wirksamkeit unter Feldexposition wurde hingegen nicht geprüft. Während der AIT wurden keine unerwünschten Reaktionen beobachtet.

Ein anderes rekombinantes Hybridprotein wurde zur AIT der Gräserpollenallergie aus einzelnen IgE-Epitoppeptiden von Wiesenlieschgrasallergenen (Phl p 1, 2, 5, 6) und der nicht allergenen PreS-Domäne des Hepatitis-B-Virus hergestellt. Dabei dient PreS als Träger für die allergenspezifischen IgE-Epitope und als ein vom Allergen unabhängiges T‑zellstimulatorisches Peptid [32, 33]. Das als BM32 bezeichnete hypoallergene Hybridprotein zeigte nach nur dreifacher (bzw. vierfacher) subkutaner Applikation an Gräserpollenallergikern keine unerwarteten systemischen, aber lokale Reaktionen an der Einstichstelle, die mit denen des Placebos vergleichbar waren. Es zeigte sich eine signifikante Symptomverbesserung (nasaler Symptomwert) in einer Allergenexpositionskammer während einer Provokation mit Gräserpollen [32]. In einer Feldstudie über 2 Graspollensaisons zeigten sich gegenüber Placebo keine signifikanten Verbesserungen im kombinierten Symptom-Medikations-Score [33]. Neben einer deutlichen Erhöhung von spezifischem IgG wurde eine reduzierte T‑Zellaktivität beobachtet.

Die AIT von Nahrungsmittelallergien stellt aufgrund der möglichen schweren allergischen Reaktionen, einschließlich Anaphylaxien, eine besondere Herausforderung dar [4]. So gibt es bis heute keine zugelassenen AIT-Präparate für die kausale Therapie von Nahrungsmittelallergien. Molekulare hypoallergene Ansätze stellen einen möglichen Beitrag zur Entwicklung neuer AIT-Ansätze für Nahrungsmittelallergien dar. Voraussetzung ist auch hier die Selektion der vorrangig relevanten Allergene. Zur AIT einer Fischallergie wurde die Allergenität des Karpfenhauptallergens Cyp c 1 durch gezielte Aminosäuresubstitution reduziert [34, 35]. Im Rahmen eines EU-geförderten Forschungsprojektes (FAST-Studie) wurde die rekombinante Faltungsmutante mCyp c 1 präklinisch und klinisch untersucht, wobei die Immunogenität bei reduzierter Allergenität (IgE-Potenz) erhalten blieb [36]. Sowohl in der First-in-man-(Phase-I/IIa-Sicherheits‑)Studie mit 15 Patienten als auch in einer multizentrischen Phase-IIb-Studie mit 41 Patienten erwies sich die hypoallergene Präparation als sehr sicher und gut verträglich. Die Wirksamkeit wurde durch die Bestimmung der Schwellendosis für Fisch mittels doppelblindem, placebokontrolliertem Food Challenge (DBPCFC) beurteilt. Das Ziel einer signifikanten Erhöhung des Schwellenwertes wurde durch die AIT nicht erreicht. Die Behandlung ging jedoch einher mit einer Induktion von IgG-Antikörpern und einer hochsignifikanten Reduktion der Hauttestreaktion auf Fisch [37].

Für die AIT der Erdnussallergie wurden hypoallergene Substitutionsvarianten der Hauptallergene Ara h 1, Ara h 2 und Ara h 3 in *E. coli* rekombinant erzeugt. Nach Inaktivierung der *E. coli*, die die Allergene einkapselten, wurden diese in einer Phase-I-Sicherheitsstudie Erdnussallergikern rektal verabreicht [38]. Jedoch traten mehrheitlich unerwünschte Reaktionen, einschließlich Anaphylaxien, auf, sodass die klinische Entwicklung beendet wurde.

## Rekombinante *Virus-like Particles* (VLP)

VLP sind multimere Strukturen, die der Morphologie eines nativen Virus entsprechen, aber nicht selbst replizieren können, da ihnen das genetische Material fehlt. Hierdurch werden Mutationen oder pathogene Infektionen ausgeschlossen. VLP bestehen aus repetitiven Proteineinheiten viraler Kapside [16]. Zugelassene oder zur Zulassung empfohlene VLP-Präparate werden in der klinischen Praxis beispielsweise zur prophylaktischen Impfung gegen humane Papillomviren (HPV; z. B. Cervarix®, Gardasil®, basierend auf HPV-Hauptkapsidprotein), Hepatitis-B-Virus (z. B. Recombivax HB®, basierend auf HBV-Oberflächenantigen) und Malaria (z. B. Mosquirix®, basierend auf Proteinen aus *Plasmodium falciparum *und HBV-Oberflächenantigen) eingesetzt. VLP stellen einen molekularen und modularen Plattformansatz dar, der es ermöglicht, Proteine, einschließlich Allergene, zu transportieren und zu präsentieren. Dabei können Allergene nach chemischer Kopplung oder als Fusionsproteine aus Allergen und Kapsidprotein auf der VLP-Oberfläche präsentiert werden. Alternativ können Allergene in VLP verpackt oder mit diesen gemischt werden. VLP haben aufgrund ihrer nanomolekularen Größe (20–200 nm) und virusähnlichen Struktur als solches immunmodulatorische Eigenschaften. Sie können zusätzlich mit adjuvantiven Elementen wie *Toll-like*-Rezeptor-(TLR‑)Liganden und T‑zellstimulatorischen Peptiden ausgestattet werden, um diese Effekte noch weiter zu verstärken [16, 39].

Bislang sind Phase-I- und Phase-II-Studien mit VLP zur Behandlung von Allergien durchgeführt worden (Tab. 2). Alle hier beschriebenen Ansätze basieren auf dem Bakteriophagen Qβ-VLP, welcher sich nach heterologer Expression (*E. coli*) des monomeren Hüllproteins von Qβ aus repetitiven Hüllproteinketten selbst assembliert. Die mittels Präzipitation und Chromatographie gereinigten VLP, welche zusätzlich mit einem synthetischen nichtmethylierten CpG-Oligodesoxynukleotid (G10) als TLR-9-Ligand gefüllt (QbG10-VLP) sind, wurden ohne Allergen [40, 41], mit Allergenextrakt gemischt [42] oder mit chemisch gekoppeltem Allergenpeptid [43] untersucht. Bereits ohne Allergen führten in einer Phase-IIb-Studie 6 subkutane wöchentliche Injektionen von QbG10-VLP im Vergleich zu Placebo zu einer signifikanten Verbesserung im kombinierten Symptom-Medikations-Score von Hausstaubmilbenallergikern mit Rhinokonjunktivitis [40]. Eine konjunktivale Provokation zeigte im Median einen zehnfachen Anstieg der Allergentoleranz [40]. Das QbG10-VLP wurde in einer weiteren Phase-II-Studie Patienten mit mildem bis moderatem allergischen Asthma bronchiale siebenmal subkutan injiziert. Im Vergleich zu Placebo zeigten sich unter kontrolliertem Steroidentzug und Verringerung der Controller-Medikation (Salbutamol) signifikante Verbesserungen in verschiedenen Parametern unter anderem im Asthma Symptom and Medication Score (ASMS) und FEV_1_ [41]. Milde bis moderate unerwünschte Reaktionen traten vornehmlich an der Injektionsstelle auf. In einer Open-Label-Phase-I/IIa-SCIT-Studie führte QbG10-VLP, mit Hausstaubmilbenextrakt gemischt, 10 Wochen nach sechsmaliger Injektion zu einer signifikanten Verringerung der allergischen/asthmatischen Symptome (erhöhte Auslösedosis im konjunktivalen Provokationstest, selbst protokollierte Symptome) von Hausstaubmilbenallergikern mit perennialer Rhinokonjunktivitis [42]. Der Effekt hielt mindestens 38 Wochen an. Schließlich wurde an QbG10-VLP ein synthetisches Epitoppeptid des Hausstaubmilbenallergens Der p 1 chemisch gekoppelt und gesunden Probanden dreimal subkutan oder intramuskulär injiziert [43]. Unabhängig von der Applikation zeigte sich eine rasche allergenspezifische Immunogenität durch Anstieg des Der p 1-spezifischen IgG bei gleichzeitig guter Verträglichkeit in den gesunden Probanden. Das Entwicklungsprogramm wurde jedoch eingestellt.

## Synthetische Peptide

Publizierte AIT-Studien mit chemisch-synthetischen hypoallergenen Peptiden (zur Herstellung [44]) liegen für die kausale Therapie von Birkenpollen‑, Katzenhaar- und Gräserpollenallergie vor (Tab. 2). Dabei wurden 2 unterschiedliche therapeutische Konzepte verfolgt. Mittels längerer, sich überlappender Fragmente werden T‑zellstimulatorische Peptide (T-Zellepitope) und weitere Sequenzabschnitte präsentiert, welche zusätzlich protektive allergenspezifische IgG induzieren sollen. Im Gegensatz dazu zielen kurze synthetische peptidimmunregulatorische T‑Zellepitope (SPIRE) insbesondere auf die Induktion von regulatorischen T‑Zellen ab, um eine Toleranz gegenüber dem Allergen herbeizuführen [18].

Ein Beispiel für lange synthetische Peptide ist ein als „AllerT“ bezeichneter Ansatz mit 3 sich überlappenden Peptiden (49–71 Aminosäuren), die die gesamte Aminosäuresequenz und das komplette T‑Zellepitoprepertoire des Birkenpollenhauptallergens Bet v 1 abdecken. Die Peptide zeigten *in vitro* keine IgE-Bindung oder Basophilenaktivierung [45]. Fünf subkutane Injektionen über 2 Monate führten bei behandelten Birkenpollenallergikern zu keinen Soforttypreaktionen, aber zu einem Anstieg von Bet-v-1-spezifischem IgG im Vergleich zu Placebobehandelten [46]. Nach 5 präsaisonalen Injektionen mit AllerT innerhalb von 2 Monaten zeigten Birkenpollenallergiker in einer placebokontrollierten SCIT-Studie eine statistisch signifikante Abnahme der kombinierten Werte von Rhinokonjunktivitissymptomatik und Medikation während der Birkenpollensaison sowie einen deutlichen IgG4-Anstieg [47]. Eine statistisch signifikante, wenn auch geringe Verbesserung der Symptomatik bestand auch während einer zweiten Birkenpollensaison [48].

Eine erste placebokontrollierte SCIT-Studie mit 2 kurzen (je 27 Aminosäuren) immunodominanten T‑Zellepitopen des Katzenhauptallergens Fel d 1 (AllervaxCAT) zeigte nach viermaliger Injektion in wöchentlichen Intervallen eine Verringerung der klinischen Symptome (Katzenexpositionsraum, nasale/pulmonale Symptomatik) von Katzenallergikern [49]. Unerwünschte allergische Episoden während der Behandlung waren vor allem respiratorische Spättypreaktionen (Brustenge, Kurzatmigkeit, Husten, Keuchen, Asthmaexazerbation), 3 Patienten der aktiven Gruppe mussten zudem systemisch mit Epinephrin behandelt werden [50]. Dieser Ansatz zur Behandlung der Katzenhaarallergie wurde mit deutlich kürzeren T‑Zellepitoppeptiden (SPIRE, 13-17 Aminosäuren) erneut aufgegriffen [51, 52]. Mit 4 intradermalen Injektionen von 7 Peptiden (ToleroMune Cat, Cat-PAD) in 4‑wöchigem Abstand zeigten Katzenhaarallergiker in einer placebokontrollierten AIT-Studie eine signifikante Verbesserung der Symptomatik (Allergenexpositionskammer (EEC), kombinierter Rhinokonjunktivitis-Symptom-Score (TRSS)), die auch nach einem Jahr noch anhielt. Behandlungsbedingte unerwünschte Reaktionen waren mehrheitlich mild [51]. Auch 2 Jahre nach Behandlung zeigte sich eine signifikante Verbesserung der Symptomatik (EEC; TRSS; [52]). Eine darauffolgende Phase-III-Studie konnte jedoch aufgrund eines sehr hohen Placeboeffekts die therapeutische Wirksamkeit nicht bestätigen [53]. Danach wurde das zugrunde liegende Therapieprogramm, das auch weitere AIT-Studien, wie beispielsweise zur Behandlung der Gräserpollenallergie [54], einschloss, vom Sponsor eingestellt.

## Regulatorische Aspekte

Wenngleich die in Tab. 2 zusammengefassten klinischen Studien nicht ausschließlich in der Europäischen Union (EU) durchgeführt wurden, beschränken wir uns in unseren regulatorischen Ausführungen auf den Geltungsbereich der EU. Innovative AIT-Präparate wie rekombinante (Hypo)Allergene, VLP und synthetische Peptide sind genau wie traditionelle extraktbasierte Therapieallergene Arzneimittel im Sinne der Europäischen Richtlinie 2001/83/EG, da sie Stoffe oder Stoffzusammensetzungen darstellen, die als Mittel zur Heilung oder zur Verhütung menschlicher Krankheiten bestimmt sind. Damit gelten die entsprechenden Vorgaben an Wirksamkeit, Sicherheit und Qualität [55]. In der Europäischen Union müssen rekombinante AIT-Präparate, im Gegensatz zu den synthetischen oder extraktbasierten Allergenprodukten, gemäß dem Anhang der Verordnung (EG) 726/2004 über ein zentralisiertes Verfahren zugelassen werden, da dies für alle Arzneimittel, die mithilfe von rekombinierter DNA in biotechnologischen Verfahren hergestellt werden, verbindlich ist [56]. Spezifische Anforderungen an die Qualität, Präklinik bzw. Klinik für die Entwicklung rekombinanter Allergene oder synthetischer Peptide werden in den entsprechenden EMA Guidelines adressiert [57, 58]. Zur Bewertung der Qualität gelten für die hier beschriebenen innovativen AIT-Produkte die Prinzipien der „Guideline on Allergen Products“ [58], nicht aber jene der Europäischen Arzneibuch Monographie über Allergenprodukte [59], da die Monografie ausschließlich für aus natürlichen Quellen gewonnene Therapieextrakte bestimmt ist. Darüber hinaus sind verschiedene „ICH-Quality Guidelines“ des „International Council for Harmonisation of Technical Requirements for Pharmaceuticals for Human Use“ anwendbar [60].

Die klinische Evaluierung von Allergenextrakten und „reinen“ Allergenen zielt immer auf die Erfüllung eines positiven Nutzen-Risiko-Verhältnisses. Bei einem therapeutischen Allergenextrakt (i.e. allergenhaltiges Proteingemisch) wird davon ausgegangen, dass alle für eine Allergietherapie relevanten Allergene enthalten sind [57]. Bei der Therapie mit „reinen“ Allergenen kann genau ausgewählt werden, welche Inhaltsstoffe im Produkt enthalten sind. Allerdings könnten solche Therapeutika eventuell nicht die bzw. nicht alle für den einzelnen Patienten relevanten allergieauslösenden Allergene beinhalten (Tab. 1). Es muss also im Vorfeld genau definiert werden, welche Allergene für die Auslösung der entsprechenden Allergie relevant sind. Allerdings können die relevanten Allergene von Patient zu Patient variieren. Entsprechend wird in der Guideline für die klinische Anwendung von Allergenprodukten gefordert, dass sowohl die Auswahl der im Therapeutikum enthaltenen Allergene als auch die zu behandelnde Patientenpopulation begründet sein muss. Dazu ist zum Beispiel die vorherige Evaluierung von individuellen Sensibilisierungsmustern der in der klinischen Studie untersuchten Patientenpopulation notwendig [57]. Dadurch soll das Risiko einer Behandlung mit einem für den individuellen Patienten nicht geeigneten Therapeutikum verringert werden. Eine auf den individuellen Patienten zugeschnittene Immuntherapie war innerhalb regulatorischer Richtlinien bisher schwierig umzusetzen, da keine patientenspezifischen komponentenaufgelösten personalisierten Rezepturen, sondern nur Fertigarzneimittel mit definierter Zusammensetzung als zulassungsfähig erachtet wurden. Daher lag das Augenmerk darauf, ein Produkt zu entwickeln und zuzulassen, welches für einen Großteil der Population erfolgreich einsetzbar ist [61]. Analog zu den aktuellen Entwicklungen von personalisierten Ansätzen zur Behandlung von Krebserkrankungen könnte sich dies zukünftig ändern [62]. Neben den verschiedenen „reinen“ Allergenpräparationen erhalten auch rekombinante VLP in der Allergologie zunehmend an Beachtung [16, 39]. Zur Bewertung können in solchen Fällen nicht ausschließlich Guidelines für Allergenprodukte angewendet werden, da diese nicht alle Aspekte abdecken. Hier müssen gegebenenfalls zusätzliche Aspekte, die für die Bewertung von chemisch-pharmazeutischen Arzneimitteln, Impfstoffen oder Adjuvanzien, soweit diese relevant sind, berücksichtigt werden: Werden beispielsweise VLP und Allergen als ein rekombinantes Fusionsprotein erzeugt, so wird dieses als aktive Substanz eingeordnet. Dabei wird das VLP selbst nicht als Adjuvans angesehen, da Adjuvanzien Hilfsstoffe sind, die getrennt von der aktiven Substanz zur Stimulierung der Immunantwort während der finalen Formulierung zugesetzt oder separat vorgelegt werden [63]. Grundlegende Anforderungen an die Entwicklung und Herstellung von Produkten aus rekombinanter DNA-Technologie werden in der Europäischen Arzneibuch Monographie über Produkte aus rekombinanter DNA-Technologie beschrieben [64].

## Fazit

Eine Vielzahl von klinischen Konzeptstudien konnte prinzipiell eine immunologische bzw. klinische Wirksamkeit (kombinierter Symptom-Medikations-Score des aktiven Präparates im Vergleich zum Placebo) von verschiedenen rekombinanten (Hypo)Allergenen, rekombinanten VLP und synthetischen Peptiden in der AIT saisonaler und perennialer allergischer Erkrankungen zeigen. Umgekehrt lässt sich aus dem molekularen AIT-Ansatz selbst keine Aussage zur Wirksamkeit ableiten. Am häufigsten wurden Studien zur kausalen Therapie von Inhalationsallergien mit einer Rhinokonjunktivitissymptomatik durchgeführt. Am Beispiel der Birkenpollenallergie zeigte sich, dass ein rekombinantes Therapieallergen eine vergleichbare Wirksamkeit (Symptom-Medikations-Score) erbringen kann wie ein traditionelles extraktbasiertes Therapieprodukt. In einzelnen Studien wurden Nahrungsmittelallergien mit unterschiedlichem Erfolg adressiert. Für die Marktetablierung bzw. Akzeptanz neuer molekularer AIT-Präparate wäre die Demonstration eines klaren Mehrwertes (beispielsweise verkürzte Therapiedauer, überlegene Wirksamkeit oder Tolerabilität) gegenüber zugelassenen extraktbasierten Therapieallergenen mit bekannter Wirksamkeit und Sicherheit förderlich. Gegebenenfalls kann dies durch Kombination und Weiterentwicklung der Erkenntnisse aus den summarisch vorgestellten Ansätzen erzielt werden und potenziell zu einer erhöhten Patientenakzeptanz und -adhärenz beitragen.
